# The effect of eggplant (Solanum melongena L.) extract peroal against blood glucose level of white rat (Ratus novergicus) wistar strain diabetic model

**DOI:** 10.1186/1687-9856-2013-S1-O33

**Published:** 2013-10-03

**Authors:** Nanda Rela Qonita, Maimun Zulhaidah, Sudiarto, Harjoedi Adji Tjahjono

**Affiliations:** 1 Faculty of Medicine, Brawijaya University, Malang, Indonesia

## 

Eggpant (Solanum melongena L.) that are common in Indonesia are already known contain anthocyanins as antioxidants as well as α-glukosidase inhibitor that can inhibit the rise of blood glucose in diabetes mellitus (DM). This study aimed to determine that the eggplant extract has antihyperglicemic effect and the effective dose as well. This was an experimental study with post test only control group design. We used 25 rats that devided into 5 groups: negative control group (PO), positive control group (PA), and 3 treatment groups (P1,P2,P3). The rats in PA, P1,P2 and P3 were injected intraperitonally with aloksan 150 mg/kgBW/day so they have blood glucose level above 200 mg/dL. Treatment group were given eggplant extract by dose P1=383 mg/kgBW/day, P2=686 mg/kgBW/day, and P3=1372 mg/kgBW/day. On day 15 after DM, the rats were terminated and the blood glucose level were measured using a sphectrophotometer. The Result of One Way ANOVA test showed that they was a significant differences between treatment groups and PA, no significant differences between P1 and P3 to PO, but there were a significant differences between P2 and PO. It can be concluded that eggplant extract may lower blood glucose level of DM rats near to normal, but the difference in dose in this study do not affect the magnitude of decrease in blood glucose level.

